# Integration analysis identifies the role of metallothionein in the progression from hepatic steatosis to steatohepatitis

**DOI:** 10.3389/fendo.2022.951093

**Published:** 2022-10-18

**Authors:** Xiaoya Li, Shaoping Zhong, Yifan Sun, Xinmei Huang, Yue Li, Lihong Wang, Yueyue Wu, Min Yang, Hai-Xin Yuan, Jun Liu, Shufei Zang

**Affiliations:** ^1^ Department of Endocrinology of Shanghai Fifth People’s Hospital, Fudan University, Shanghai, China; ^2^ Department of Neurology, Zhongshan Hospital, Fudan University, Shanghai, China

**Keywords:** non-alcoholic fatty liver disease, steatohepatitis, metallothionein, integration analysis, robust rank aggregation

## Abstract

**Background:**

Non-alcoholic fatty liver disease (NAFLD), a metabolic disorder that develops from non-alcoholic fatty liver (NAFL) to non-alcoholic steatohepatitis (NASH), has become an epidemic of chronic liver dysfunction worldwide. However, mechanisms that govern the transition from NAFL to NASH have not been fully elucidated.

**Methods:**

Gene expression profile data of NAFLD liver tissues were obtained from Gene Expression Omnibus (GEO), including three microarray datasets with 60 NAFL and 44 NASH patients. Integrative differentially expressed genes (DEGs) between NAFL and NASH patients were identified using robust rank aggregation (RRA) analysis. Hub genes were identified combined with gene ontology functional annotation and protein–protein interaction network construction and validated using a sequencing dataset. Huh-7 cells with palmitate-induced lipid overload and NAFLD-diet mouse model of different stages were used to verify our findings.

**Results:**

RRA analysis determined 70 robust DEGs between NAFL and NASH. The most robustly upregulated genes were *SPP1*, *AKR1B10*, *CHST9*, and *ANXA2*, while the most robustly downregulated DEGs were *SNORD94*, *SCARNA10*, *SNORA20*, and *MT1M*. Cellular response to zinc ion (GO: 0071294) ranked first in GO analysis of downregulated genes, and Kyoto Encyclopedia of Genes and Genomes (KEGG) enrichment showed that mineral absorption (hsa04978) was significantly enriched. The involvement of the metallothionein pathway was further validated by the decrease of *Mt1* expression during NAFL to NASH progression in NAFLD mice and the protection from lipotoxicity in liver cells by overexpressing MT1M.

**Conclusions:**

Our integrated analysis identified novel gene signatures and provided comprehensive molecular mechanisms underlying the transition from NAFL to NASH. Metallothionein might be a potential intervention target for NAFLD progression.

## Highlights

Our findings provide novel gene signatures of NASH progression and novel evidence that MT1 exacerbates the progression of NASH. Clinically, reduced expression of MT1 may become a potential diagnostic marker, and restoring the function of MT1 might be a potential intervention target for blocking NASH progression.

## Introduction

Non-alcoholic fatty liver disease (NAFLD) remains one of the most important causes of chronic liver disease. Paralleling the worldwide increase in obesity, the global prevalence of NAFLD is currently estimated to be 24% (1). NAFLD is a comprehensive term that comprises a series of consecutive liver conditions varying in the severity of the injury. Among these, hepatic steatosis alone is referred to as non-alcoholic fatty liver (NAFL), while non-alcoholic steatohepatitis (NASH) is defined as a more serious process characterized by inflammation, hepatocyte damage, and pericellular fibrosis, which may progress to cirrhosis (2).

Although NAFL or NASH can be strongly suspected in an individual on the basis of imaging and clinical features (such as the presence of metabolic comorbidities and abnormal lab results), their definite diagnosis still depends on liver biopsy. Patients with only NAFL have a relatively benign course and are principally reversible, whereas the presence of NASH carries a poor prognosis and increases the risks of adverse outcomes including cirrhosis, liver failure, hepatocellular carcinoma, and even non-liver-associated adverse outcomes (cardiovascular disease and malignancy) (3). Moreover, NASH is now considered one of the most common indications for liver transplantation worldwide. Although steady progress has been made in clarifying the pathogenesis, identifying therapeutic targets, and developing novel drugs for NAFLD, there is still inadequate knowledge about the pathogenic drivers from NAFL to NASH. It is noteworthy that, based on longitudinal studies, one-third of patients with hepatic steatosis are estimated to progress to steatohepatitis (4). In current standard clinical practice, non-invasive serum and imaging markers are not enough to distinguish relatively benign NAFL from progressive steatohepatitis. Also, the validation of predictive disease progression risks and treatment effects is lacking and urgently needed.

Given the rapid growth of NAFLD and irreversible outcomes, it is important to elucidate the underlying mechanisms that mediate the pathological process from NAFL to NASH. Identifying gene-specific expression patterns has been proven useful in the understanding of pathogenic mechanisms or therapeutic assessment for NAFLD. In the past decades, microarray has been widely used for gene expression profiling in liver tissues from NAFLD patients of different stages. However, there is great heterogeneity among the findings of those microarray studies, partially due to the employment of different analysis platforms, data outliers, and populations (5). Robust rank aggregation (RRA), as a newly developed integrative bioinformatics method, has been widely used to define robustly integrated disease-related mRNA profiles from multiple microarray studies in various diseases. To our knowledge, the RRA method has not been used in previous studies to identify differentially expressed genes (DEGs) from NAFL to NASH progression, which inspired us to conduct this research.

In the present study, we performed a meta-analysis of gene expression between NAFL and NASH liver tissues integrating multiple microarray datasets from the Gene Expression Omnibus (GEO) database. Hub gene identification in the DEGs through RRA analysis, gene enrichment, pathway annotation, and protein–protein interaction (PPI) analysis were also performed. We also used sequencing data (GSE126848) to validate hub DEGs from integration analysis. Then we further investigated the function of metallothionein, the most robustly downregulated gene family, in the progression of NAFLD. We evaluated the expression of *Mt1* in the NASH mouse model and *MT1M* in a lipid-overload hepatocellular model and proved that overexpression of MT1M protects liver cells from lipotoxicity. Our study demonstrated hitherto unknown molecular signatures from NAFL to NASH and suggested that the metallothionein pathway might be a potentially effective therapeutic target for NAFLD progression.

## Methods

### Microarray datasets and differentially expressed gene identification

Microarray datasets were searched using the following terms: “Non-alcoholic”, “Fatty liver”, “Steatohepatitis”, “Gene expression”, “Homo sapiens”, and “Microarray” systematically. Datasets were included according to the following criteria: 1) containing at least 30 total samples, 2) patients were pathologically diagnosed through liver biopsy, 3) both NAFL and NASH patients were included and clearly distinguished, 4) and raw data or gene expression profiling by array were available in GEO. Expression profile matrix files and related annotation documents of three datasets (GSE48452, GSE89632, and GSE66676) were downloaded from the GEO database (https://www.ncbi.nlm.nih.gov/gds/). The detailed information of these datasets is shown in **Table 1**. Microarray probes were mapped with gene symbols through the corresponding annotation document. All datasets were standardized by quantiles. Limma R package was used to screen for DEGs in each dataset. Genes with a corrected *p*-value <0.05 and |log_2_ fold change (FC)| > 0.58 were considered as DEGs.

**Table 1:** Details of the GEO NAFLD data.

GSE ID	Numbers of liver samples	GPL ID	Country	Citation (PMID)	Time	Diagnostic criteria
GSE48452	14 NAFL and 18 NASH	GPL11532	Germany	23931760	2013	Kleiner et al., 2005
GSE89632	20 NAFL and 19 NASH	GPL14951	Canada	25581263	2016	Kleiner et al., 2005
GSE66676	26 NAFL and 7 NASH	GPL6244	USA	26026390	2017	Brunt EM et al., 2011

The diagnostic criteria from the two cited articles are the same; that is, the pathology score greater than 5 is diagnosed as “NASH”.

GEO, Gene Expression Omnibus; NAFLD, non-alcoholic fatty liver disease; NAFL, non-alcoholic fatty liver; NASH, non-alcoholic steatohepatitis.

1. GSE48452: Experiment type: Expression profiling by array.

GPL11532 [HuGene-1_1-st] Affymetrix Human Gene 1.1 ST Array [transcript (gene) version].

2. GSE89632 Expression profiling by array.

GPL14951 Illumina HumanHT-12 WG-DASL V4.0 R2 expression beadchip.

3. GSE66676 Expression profiling by array.

GPL6244 [HuGene-1_0-st] Affymetrix Human Gene 1.0 ST Array [transcript (gene) version].

### Comprehensive analysis by robust rank aggregation method

The “Robust Rank Aggregation” R package was used to integrate the ranked gene lists of each dataset as previously reported (6). Genes with *p*-value <0.05 and |log_2_ FC| > 0.58 were considered as robust DEGs.

### Gene ontology functional enrichment, Kyoto Encyclopedia of Genes and Genomes pathway analysis, and protein–protein interaction network construction

DAVID provides a comprehensive set of functional annotation tools for investigators to understand the biological meaning behind a large list of genes (7). To further analyze the results from RRA integration, we submit the list of robust DEGs to the website (https://david.ncifcrf.gov/) for gene ontology (GO) functional enrichment and Kyoto Encyclopedia of Genes and Genomes (KEGG) pathway analysis. HIPPIE database (http://cbdm.uni-mainz.de/hippie/) was used to perform the PPI network analysis, and a confidence score greater than 0.4 in the PPI network was considered significant.

### Hub gene identification and validation by sequencing data

Hub genes were identified by ranking nodes in the PPI network using cytoHubba, a plugin of Cytoscape. To validate the reliability of hub genes, a sequencing dataset (GSE126848, including 15 NAFL and 16 NASH patients) was analyzed using the edgeR package. Genes with *p*-value <0.05 were considered significant.

### Animals and tissue preparation

Male C57BL/6J mice were obtained from Shanghai SLAC Laboratory and carefully housed under pathogen-free conditions. Mice were fed with a NASH diet containing 40% fat (of which 18% was trans-fat), 22% fructose, and 2% cholesterol (D09100301, Research Diets Inc., New Brunswick, NJ, USA) or a normal diet for 16 or 20 weeks. Mice were fed with a choline-deficient, l-amino acid-defined, high-fat diet with a 0.1% methionine (CDAHFD) diet (A06071302, Research Diets Inc.) or a control diet (A06071314, Research Diets Inc.) for 6 weeks. All animal-related experimental procedures were performed in accordance with the National Institutes of Health guidelines and approved by the Laboratory Animal Ethical Committee of Fudan University. After being fed separately, the mice were sacrificed for analysis. Just before sacrifice, blood was drawn directly from the heart, and the serum was separated and stored at −80°C. Liver tissues were rapidly harvested and fixed in 4% paraformaldehyde in phosphate-buffered saline (PBS) or snap-frozen in liquid nitrogen and stored at −80°C for later assessment.

### Liver function parameters analysis and non-alcoholic fatty liver disease activity scoring

Serum alanine transaminase (ALT) and aspartate transferase (AST) levels were determined using commercial enzymatic kits (Roche, Basel, Switzerland) by an automatic biochemical analyzer (Roche cobas C702). The total non-alcoholic fatty liver disease activity score (NAS) represents the sum of scores for steatosis, lobular inflammation, and hepatocyte ballooning according to hematoxylin and eosin (H&E)-stained liver sections. NAS ranged from 0 to 8, which was calculated by a sum of scores of steatosis (0–3), lobular inflammation (0–3), and hepatocyte ballooning (0–2) according to criteria set forth by a well-validated grading system. The definition of NASH corresponded to a diagnosis of definite NASH according to the NASH Clinical Research Network Scoring System. Samples were blindly scored by two experienced pathologists.

### Determination of liver triglyceride content

Hepatic triglyceride (TG) was determined by using the tissue TG assay kit (Applygen, Beijing, China) following the manufacturer’s instructions. Briefly, the experimental procedure was carried out as follows. Approximately 50 mg of liver samples was cut, homogenized using a homogenizer, and lysed in lysis buffer for 10 min. After centrifugation at 2,000 × *g* for 5 min, the supernatants were divided into two parts: one for TG measurements and the other for protein concentration. Protein quantitation was conducted using the bicinchoninic acid (BCA) protein assay kit (P0011, Beyotime, Shanghai, China). The final TG levels were standardized by protein concentration.

### H&E staining

Samples from the right lobe of all mouse livers were fixed in 4% paraformaldehyde for at least 24 h at room temperature, embedded in paraffin, and sectioned into 8-μm tissues. Slices were stained with Mayer’s H&E (Baso, New Taipei, Taiwan). Images were captured from three randomly selected fields using Olympus BX61VS.

### RNA isolation and RT-PCR

Total RNA from mouse livers was extracted using TRIzol and reverse transcribed using TransScript One-Step gDNA Removal and complementary DNA (cDNA) Synthesis SuperMix (TransGen Biotech, Beijing, China). The resulting cDNAs were used for PCR using the TB Green^®^ Premix Ex Taq™ (Takara, Dalian, China). The relative abundance of mRNA was determined on a 7500 real‐time PCR system (Thermo Fisher Scientific, Waltham, MA, USA). The relative gene expression for all genes was analyzed using the 2^−ΔΔCt^ method by normalizing with ACTIN gene expression in all experiments and analyzed by Student’s t-test or one-way analysis of variance (ANOVA). The primer pair sequences used for RT-PCR reactions for *in vivo* studies are listed in **Supplementary Table 3**.

### Cell culture and treatment

Huh-7, a human hepatoma cell line, was obtained from ATCC and cultured in Dulbecco’s modified Eagle’s medium (Gibco, Grand Island, NY, USA) supplemented with 10% fetal bovine serum (Gibco) and 50 μg/ml of penicillin/streptomycin. Unsaturated fatty acids, such as palmitic acid, are believed to agitate the progress of steatohepatitis by inducing cellular stress and organelle toxicity of fatty acids. Several studies have elucidated that exposure to palmitic acid activates inflammatory and apoptotic pathways (8); other studies also indicated that it induced lipo-apoptosis and chemokine secretion (9). Therefore, we treated Huh-7 cells with palmitic acid to mimic the lipotoxicity underlying NASH pathological progression. Palmitic acid complexed with bovine serum albumin (BSA) was made as follows: palmitic acid powder was added to a 10% solution of fatty acid-free BSA (Sigma, St. Louis, MO, USA) and dissolved by shaking gently overnight at 37°C to yield a 25-mM solution.

### Lentiviral plasmid production and infection

Full-length cDNA of MT1M was cloned into the pCDH vector using standard protocols. To generate Huh-7 cells with MT1M overexpression (MT1M-OE), lentivirus was produced by transfecting pCDH vector or pCDH-MT1M together with psPAX2 and pMD2G packaging plasmids into HEK293T cells. Lentiviral supernatant was harvested 36 h after transfection and cleared by a 0.45-μM filter, which was then used to infect Huh-7 cells after being supplemented with polybrene (8 μg/ml). Stable cell pools were selected with puromycin (1 μg/ml, Amresco, Solon, OH, USA) for 3 days.

### Statistical analysis

Results were analyzed using GraphPad Prism 8.0. Statistical analyses were performed using a two-tailed unpaired Student’s t-test or one-way ANOVA with multiple comparisons as indicated in corresponding figure legends.

## Results

### Screening differentially expressed genes between non-alcoholic steatohepatitis and non-alcoholic fatty liver from microarray data

**Figure 1:**
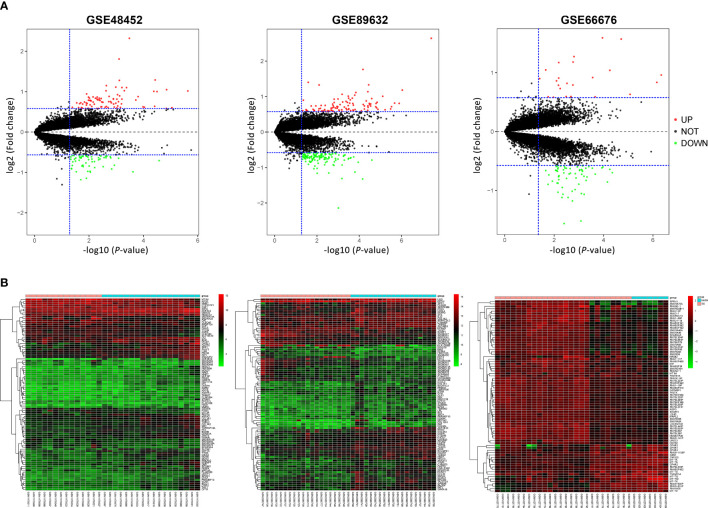
Volcano plots and heatmap of three microarray datasets. **(A)** Volcano plots of GSE48452, GSE89632, and GSE66676. Red points represent upregulated genes, and green points represent downregulated genes. **(B)** Heatmaps of GSE48452, GSE89632, and GSE66676, and red represents upregulated genes while blue represents downregulated genes in NASH compared with NAFL patients. NAFL, non-alcoholic fatty liver; NASH, non-alcoholic steatohepatitis.

According to previously established inclusion criteria, GSE48452, GSE66676, and GSE89632 were included in this study. A total of 104 NAFLD patients (60 patients with NAFL and 44 patients with NASH) were analyzed. Before screening for DEGs in NAFLD, the expression profile was normalized to ensure comparability between datasets, and the results are shown in **Supplementary Figure 1**. Limma R package was used to screen DEGs, and the volcano plots and cluster heatmaps in **Figure 1** covered DEGs from the above datasets.

### Identification of integrated differentially expressed genes

**Figure 2:**
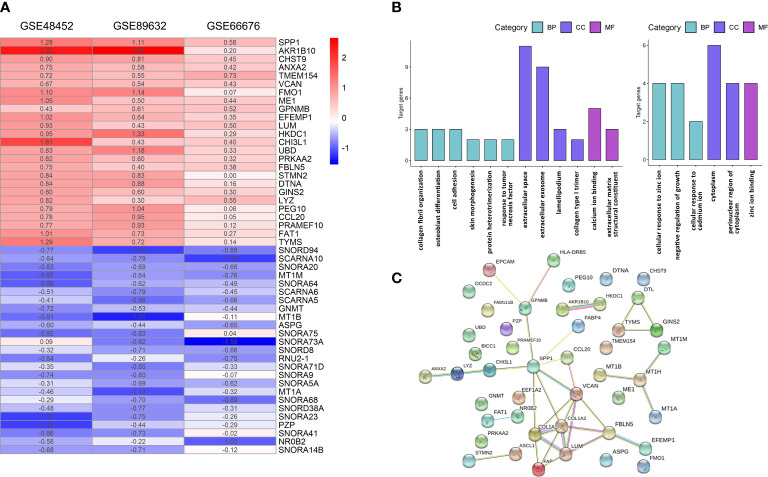
Integrative analysis of DEGs by RRA, functional enrichment, and PPI network. **(A)** Heatmap of the top 25 robustly upregulated or downregulated DEGs in RRA analysis. **(B)** GO annotation of upregulated (left panel) and downregulated (right panel) DEGs. **(C)** PPI networks of DEGs. BP, biological process; CC, cellular component; GO, gene ontology; MF, molecular function; PPI, protein–protein interaction; DEGs, differentially expressed genes; RRA, robust rank aggregation.

After normalization and quality control, integrated DEGs were screened using the RRA method, which assumes that each gene is randomly ordered in each dataset. The smaller the *p*-value in the RRA results, the higher ranks and robustness of gene differential expression. A total of 70 robustly integrated DEGs were identified, and their detailed *p*-value and log_2_FC are shown in **Supplementary Table 1**. The top 25 upregulated and downregulated integrated genes are shown in **Figure 2A**, and heatmap visualized the top five upregulated genes (secreted phosphoprotein 1 (SPP1; *p*-value = 3.38E−09), AKR1B10 (*p*-value = 1.24E−08), CHST9 (*p*-value = 8.79E−08), ANXA2 (*p*-value = 1.88E−07), and TMEM154 (*p*-value = 2.66E−07)) and the top downregulated genes (SNORD94 (*p*-value = 1.64E−09), SCARNA10 (*p*-value = 1.14E−08), SNORA20 (*p*-value = 9.45E−08), MT1M (*p*-value = 2.40E−07), and SNORA64 (*p*-value = 3.08E−07)).

### Functional annotation, protein–protein interaction network analysis, and identification of hub genes

The functional GO annotation analysis showed that the upregulated DEGs in NASH were enriched for extracellular space, collagen fibril organization, extracellular matrix structural constituent, collagen type I trimer, and lamellipodium compared to NAFL. In terms of downregulated DEGs, cellular response to zinc ions was the most significantly enriched GO term (**Figure 2B**). Detailed results of GO enrichment analysis are shown in **Supplementary Table 2**. As for KEGG pathway analysis, upregulated DEGs were significantly enriched in five pathways, including the PI3K-Akt signaling pathway and fructose and mannose metabolism. The downregulated DEGs only showed great enrichment in mineral absorption (**Table 2**). HIPPIE online database was used to perform a PPI network analysis of the DEGs, and the connections between nodes were visualized to identify the interactions between proteins translated by DEGs (**Figure 2C**). Identification of hub genes located in the central node is expected to play an important role in understanding the biological mechanism of NAFLD progression. With the use of Cytoscape, the nine most critical genes were finally defined as hub genes (*COL1A2*, *VCAN*, *COL1A1*, *LUM*, *SPP1*, *FBLN5*, *FAP*, *MT1M*, and *GPNMB*) based on the results of RRA analysis and PPI network.

**Table 2:** KEGG analysis of integrated DEGs.

Enriched KEGG pathway with upregulated genes				
Pathway	ID	Count	Genes	*p*-Value
Receptor interaction	hsa04512	3	COL1A2, COL1A1, SPP1	1.34E−02
PI3K-Akt signaling pathway	hsa04151	4	COL1A2, COL1A1, PRKAA2, SPP1	2.85E−02
Fructose and mannose metabolism	hsa00051	2	AKR1B10, HKDC1	6.27E−02
Galactose metabolism	hsa00052	2	AKR1B10, HKDC1	6.27E−02
Focal adhesion	hsa04510	3	COL1A2, COL1A1, SPP1	6.47E−02
**Enriched KEGG pathway with downregulated genes**				
**Pathway**	**ID**	**Count**	**Genes**	***p*-Value**
Mineral absorption	hsa04978	4	MT1M, MT1A, MT1B, MT1H	4.82E−06

KEGG, Kyoto Encyclopedia of Genes and Genomes; DEGs, differentially expressed genes.

### Validation of hub genes by sequencing data

**Figure 3:**
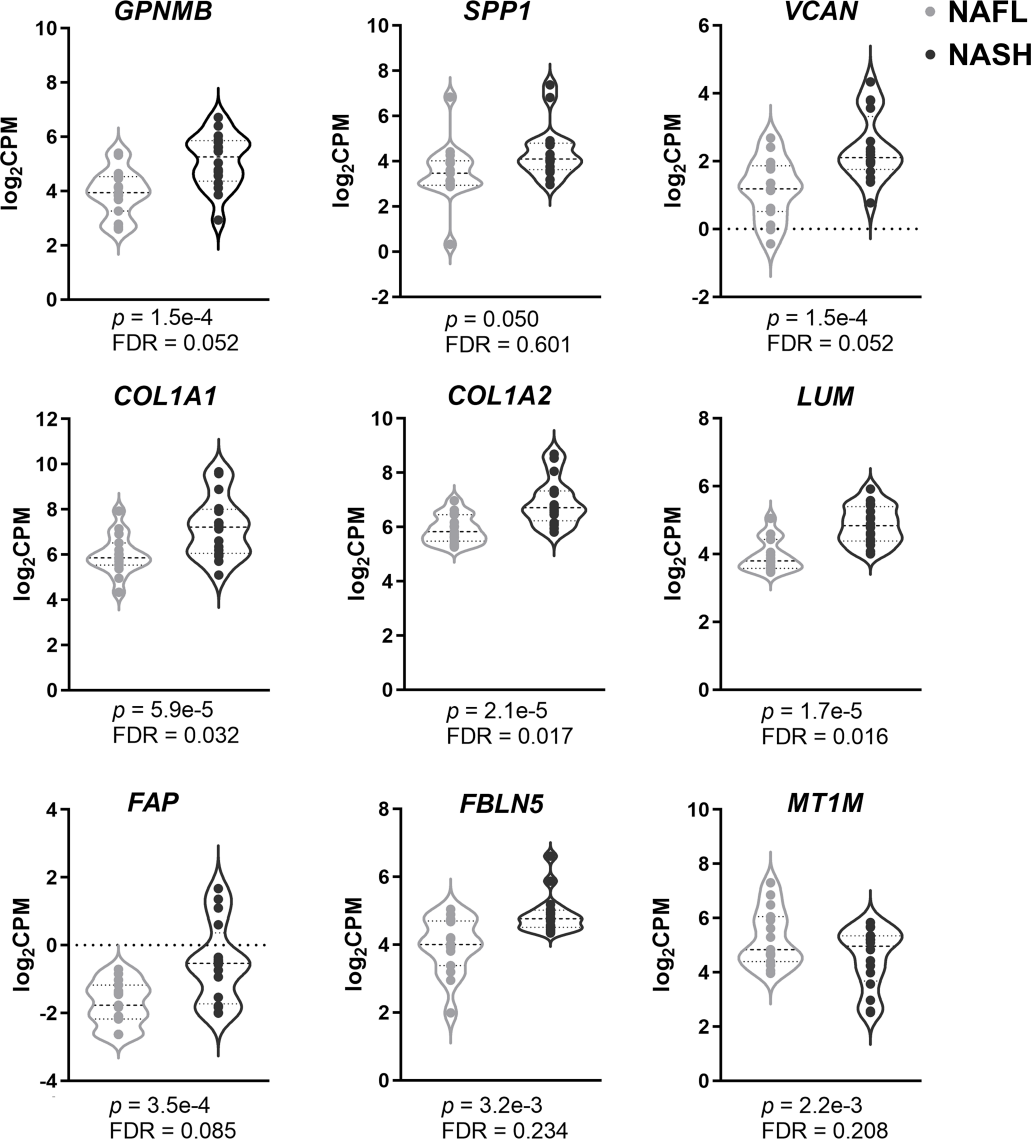
Validation of hub genes using sequencing data. Violin plots of the nine hub genes’ expression (GPNMB, SPP1, VCAN, COL1A1, COL1A2, LUM, FAP, FBLN5, and MT1M) between NASH and NAFL. Genes with *p*-value <0.05 were considered to be significant. FDR, false discovery rate; CPM, counts per million; NASH, non-alcoholic steatohepatitis; NAFL, non-alcoholic fatty liver.

To further validate the credibility of these hub genes, we examined them in a sequencing dataset (GSE126848). **Figure 3** shows that all hub genes exhibited the same variation tendency in GSE126848 as in RRA results. GPNMB, SPP1, VCAN, COL1A1, COL1A2, LUM, FAP, and FBLN5 were significantly upregulated, while MT1M was the only downregulated hub gene in NASH compared with NAFL. We also verified the expression levels of these genes between healthy and NAFL groups (GSE48452, GSE89632, and GSE66676) (**Supplementary Figure 2**). Interestingly, the expression of several hub genes (i.e., VCAN, FBLN5, and MT1M) had no difference between healthy and NAFL livers, which suggests that their alterations may be specific to the later stages of NAFLD (from NAFL to NASH).

### Decreased *Mt1* expression during non-alcoholic fatty liver to non-alcoholic steatohepatitis transition in non-alcoholic fatty liver disease mice

Given that MT1M, an encoding gene of metallothionein, is the only downregulated hub gene identified and did not alter at the early stages of NAFLD, we further examined it in a NAFLD model. As previously described, chronic administration of a high-fat, high-fructose, and high-cholesterol diet (NASH diet) is a proven approach to replicate the pathological progression from hepatic steatosis to steatohepatitis in C57BL/6J mice, with 16-week feeding mimicking the NAFL stage and 20-week feeding mimicking the NASH stage approximately (10).

**Figure 4:**
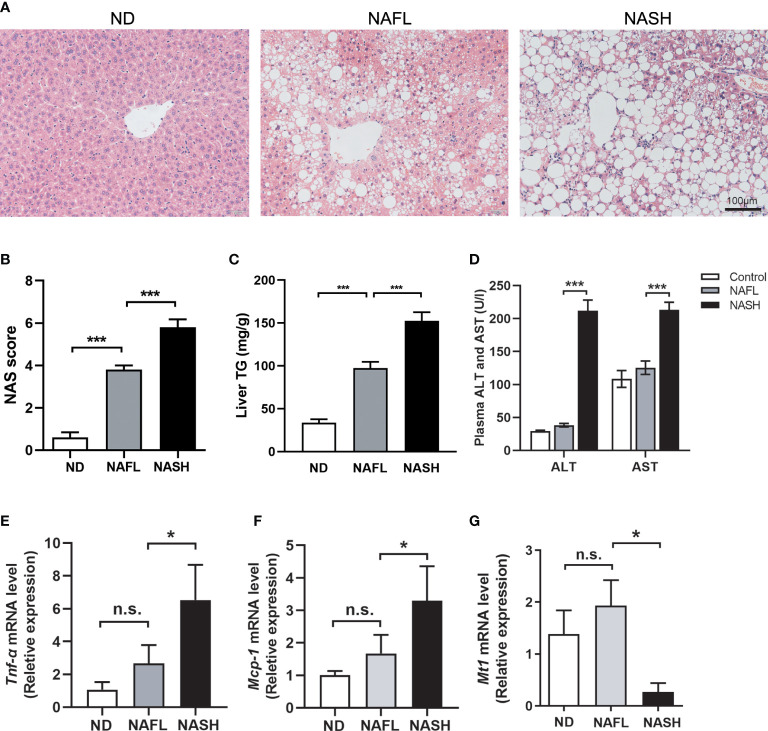
Decreased expression of *Mt1* in NASH mouse model. **(A)** H&E staining of ND, NAFL, and NASH mice. **(B)** Liver TG quantification of ND, NAFL, and NASH mice. **(C)** NAS score assessed in H&E-stained liver sections. **(D)** Serum ALT and AST of three groups of mice. **(E, F)** Expression of *Tnfα* and *Mcp1* measured by RT-PCR from ND, NAFL, and NASH mice. **(G)** Expression of *Mt1* measured by RT-PCR from ND, NAFL, and NASH mice. The expression level of genes was standardized by ND group in **(D, E)** N = 5 mice per group, and all data are shown as mean ± SEM. *p < 0.05, ***p < 0.001. “n.s.” means no significance. TG, triglyceride; NASH, non-alcoholic steatohepatitis; ND, normal diet; NAFL, non-alcoholic fatty liver; NAS, non-alcoholic fatty liver disease activity score; ALT, alanine transaminase; AST, aspartate transferase.

Thus, mice were grouped into three groups: ND (normal diet), NAFL (16-week NASH diet), and NASH (20-week NASH diet). H&E staining showed an accumulation of lipid droplets in livers of the NAFL group, which was exaggerated and accompanied by obvious inflammatory cell infiltration during prolonged 20-week feeding in the NASH group (**Figure 4A**). More representative images from four different mice for each group are shown in **Supplementary Figure 3**.

Quantification of TG extracted from livers in a colorimetric assay shows increased TG content in NAFL and NASH livers (**Figure 4B**). NAS, a diagnostic criterion of liver steatohepatitis, was significantly increased in NASH compared with the NAFL group (**Figure 4C**). It is notable that serum ALT and AST elevations were detected in the NASH group instead of the NAFL group (**Figure 4D**), consistent with the clinical fact that liver damage is frequently absent in the early stages of NAFLD. The expression of liver inflammatory genes, including *Tnf-*α and *Mcp-1*, was also upregulated in the NASH group compared to the NAFL group (**Figures 4E, F**). All of these suggested that our NAFLD model could well replicate the key aspects of different stages in NAFLD. In humans, metallothioneins are encoded by 17 genes from four families (MT1–MT4). MT1 is the largest gene family containing 13 members, including MT1M. However, in rodents, there is only one member of the *Mt1* family (11). Therefore, we quantified the expression level of *Mt1* in our NAFLD model through RT-PCR. Consistent with our findings in human datasets, the expression of *Mt1* was significantly decreased in the liver tissues of the NASH group compared to the ND and NAFL groups (**Figure 4G**). This suggested that *Mt1* downregulation might participate in the pathological progression from NAFL to NASH in mice.

### Improvement of non-alcoholic steatohepatitis by overexpressed Mt1 in mouse liver

**Figure 5:**
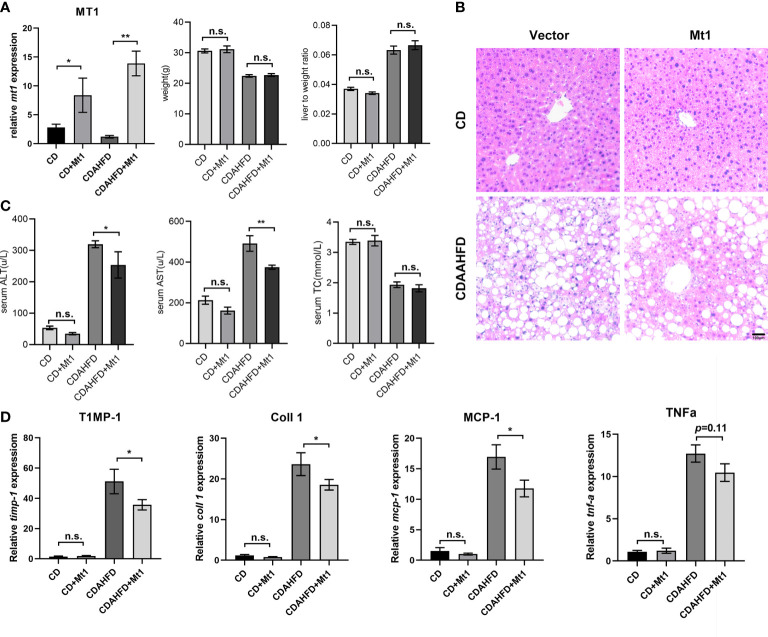
MT1 improves liver fibrosis in CDAAHFD mouse model. **(A)** Expression of Mt1 measured by RT-PCR and weight, and liver-to-weight ratio of each group of mice. **(B)** H&E staining of liver sections. **(C)** Serum ALT, AST, and TC of four groups of mice. **(D)** Expression of *Timp-1*, *Coll1*, *Mcp1*, and *Tnfα* measured by RT-PCR from CD, CD+Mt1, CDAHFD, and CDAHFD+Mt1 mice. N = 5 mice per group, and all data are shown as mean ± SEM. *p < 0.05, **p < 0.01. “n.s.” means no significance. ALT, alanine transaminase; AST, aspartate transferase; TC, total cholesterol.

In order to identify the potential intervention role of metallothionein, we overexpressed mt1 through adeno-associated virus (AAV) to verify its protective function based on an improved CDAHFD model. The dietary methionine/choline-deficient model in mice caused severe weight loss and liver atrophy, while several months of a high-fat diet did not affect fibrosis. A previous study combined the two dietary patterns and proposed a modified CDAHFD model (12) that rapidly developed progressive liver fibrosis. Therefore, we fed mice with a 6-week CDAHFD diet. The experimental mice were divided into four groups (CD, CD+mt1, CDAHFD, and CDAHFD+mt1) with five to seven mice per group to study the effect of metallothionein 1 on CDAHFD-induced liver injury in mice. Through tail veil injection, MT1 was overexpressed in the mouse liver, and there is no difference in weight and liver-to-weight ratio (**Figure 5A**; **Supplementary Figure 4**). Morphological staining showed that the degree of steatosis in the CDAHFD+mt1 group was slightly lighter than that in the CDAHFD group, and hepatic AST had improved in the CDAHFD+mt1 group (**Figures 5B, C**). Moreover, inflammation and fibrosis-related genes (timp-1, coll1, ten-α, and mcp-1) were downregulated or had a tendency of downregulation (**Figure 5D**), which suggested that overexpression of MT1 could partially alleviate the development of liver fibrosis.

### Protection of liver cells from lipotoxicity by MT1M overexpression

**Figure 6:**
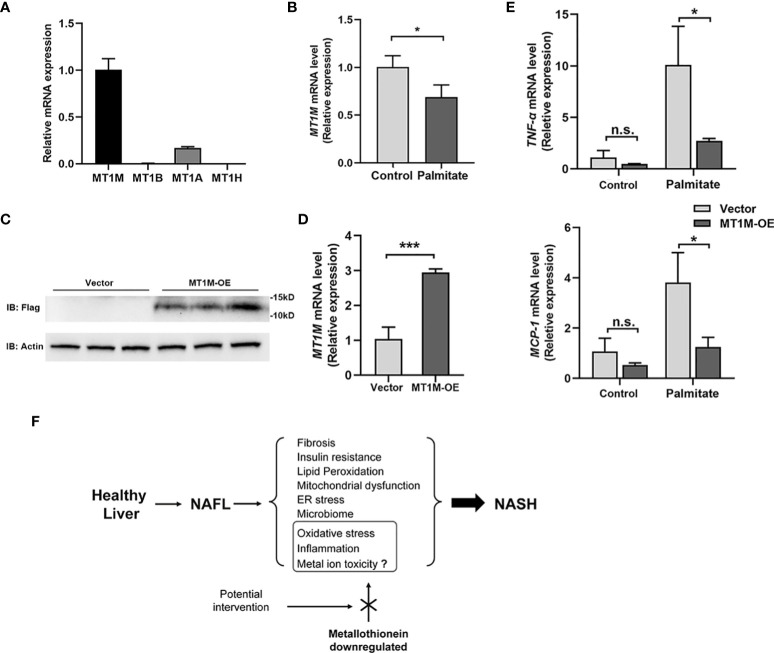
MT1M inhibits inflammation in Huh-7 cell exposure to palmitic acid. **(A)** The endogenous gene expression of MT1M, MT1B, MT1A, and MT1H in Huh-7 cells. **(B)** MT1M expression of Huh-7 cells treated with palmitate (250 μM, 12 h). **(C)**Verification of MT1M over-expression (MT1M-OE)efficiency in Huh-7 cells by western blot. **(D)** Verification of MT1M-OE efficiency in Huh-7 cells by RT-PCR. **(E)** Expression of *TNFα* and *MCP1* was measured by RT-PCR from vector or MT1M-OE Huh-7 cells treated with or without palmitate (250 μM, 12 h). Data are shown as mean ± SEM. *p < 0.05, ***p < 0.001. “n.s.” means no significance. **(F)** Proposed working model of MT in the regulation of NASH progression. MT, metallothionein; NASH, non-alcoholic steatohepatitis.

Four members of MT1 gene family (*MT1M*, *MT1B*, *MT1A*, and *MT1H*) were identified as robustly downregulated DEGs in our RRA analysis, among which *MT1M* ranked the first. Impressively, *MT1M* is also the highest expressed gene in Huh-7 cells among them (**Figure 6A**). Upon palmitic acid treatment, the expression of *MT1M* decreased in Huh-7 cells (**Figure 6B**). We further overexpressed MT1M in Huh-7 cells (**Figures 6C, D**) and found that inflammatory genes (*TNF-α* and *MCP-1*) were significantly suppressed by MT1M overexpression as compared to cells without overexpression under palmitate treatment (**Figure 6E**). Taken together, these data reinforced the notion that MT1M plays a protective role during the transition from NAFL to NASH (**Figure 6F**).

## Discussion

NASH is characterized by accumulating defects in cellular organelles, elevated levels of cytokines, recruitment of macrophages, and subsequent changes leading to the remodeling process of the intracellular matrix, which paves the way to fibrosis and possibly cirrhosis. However, NASH-related complications rarely occur at NAFL, an earlier stage of NAFLD characterized by steatotic hepatocytes that are incapable of disposing incoming lipids. The initially proposed “two-hit” model by Day and James provides a pathophysiologic rationale for the progression to steatohepatitis, claiming that the reversible intracellular deposition of triacylglycerols (“first hit”) leads to metabolic and molecular alterations and sensitizes the liver to the “second hit”, usually referred to oxidative stress and cytokine-induced liver injury (13). During the following decades, a large number of studies intended to investigate intrinsic mechanisms of NAFL-NASH progression and new targets to relieve the progression. However, a small number of genes were reported differentially expressed between NAFL and NASH, most of which were correlated with the severity and varying degrees of inflammation (14). It remains unclear which factor triggers the disequilibria between pro- and anti-inflammatory pathways, ultimately turning a benign stage of steatosis into a progressive condition.

With the large amounts of data generated by high-throughput technologies, the integration of additional biological information is of great importance to reveal the pathogenesis underlying the progression from NAFL to NASH. In this study, we used the RRA method, a well-designed tool with strong robustness to noise and high computational efficiency (6), to identify the DEGs in the switch from NAFL to NASH. Although a recent study revealed cancer-related molecular signatures in hepatic steatohepatitis but not in steatosis, it included both alcoholic and non-alcoholic fatty liver samples (15), which might cause deviations in results because of the introduction of confounding factors (alcohol).

In the present study, a total of 70 robust DEGs were identified through RRA integrative analysis from three datasets containing patients with NAFL or NASH. Functional annotation, PPI network construction, and sequencing data validation were further performed to understand the potential biological function of these DEGs. Nine of them were identified as hub genes, including *GPNMB*, *SPP1*, *VCAN*, *COL1A1*, *COL1A2*, *LUM*, *FAP*, *FBLN5*, and *MT1M*. *GPNMB* is one of the most highly expressed genes at the early and intermediate stages of eosinophil development, which plays an anti-inflammation function in NAFLD progression. A recent study has shown that GPNMB was highly expressed in a dietary NASH model and may serve as a specific biomarker for NASH (16). SPP1, also named osteopontin, was known as a profibrogenic extracellular matrix protein and Hedgehog pathway-induced cytokine. As SPP1 is involved in fibrosis regulation and triggers the release of multiple inflammatory cytokines, its activity is increased in NAFLD and paralleled with fibrosis stages, and SPP1 neutralization might be useful for preventing progressive hepatic fibrosis in NASH patients (17). VCAN, a member of the versican proteoglycan family, plays a role in the extracellular matrix and intercellular signaling and is closely associated with tumorigenesis (18). COL1A1 and COL1A2 are the major components of type I collagen, a kind of fibril-forming collagen (19). LUM, a member of the small leucine-rich proteoglycan family, is involved in collagen binding and extracellular matrix structural constituent (20). Moreover, FBLN5 is essential for elastic fiber formation, as it involves the assembly of continuous elastin polymer and promotes the interaction of microfibrils and elastin (21). Since the formation of hepatic fibrosis is a complex process of multi-factor and multi-cell involvement, our findings also point to the possible involvement of VACN, COL1A1, COL1A2, LUM, and FBLN5 in the generation of fibrotic response from NAFL to NASH. FAP is a serine protease belonging to an S9B prolyl-oligo-peptidase subfamily, which has been reported to regulate the degradation of metabolic hormone FGF21 in obese mice to provide robust metabolic benefits (22). All these hub genes are associated with inflammatory cytokines release and fibrosis formation and have been reported involved in NAFLD progression, indicating that the integrative analysis method we used is of great reliability.

*MT1M* was identified as the only downregulated hub gene in this study. In addition, metallothionein (MT)-related pathways (i.e., zinc ion and mineral absorption) were also enriched in enrichment analysis, inspiring us to focus on the role of the MT family in the progression from NAFL to NASH. MT family is composed of short cysteine-rich proteins involved in metal metabolism and detoxification and ubiquitously expressed in various tissues with the highest level in the liver. They protect cells against free radicals, oxidative stress, and damage caused by electrophilic carcinogens and thereby constitute a critical surveillance system against carcinogenesis (23). Notably, none of MT family genes was identified in a previous integrated study between healthy and NAFLD livers (24). Based on pathological immunostaining of liver biopsy samples from 37 patients, a previous study reported that MT1/2 levels were significantly reduced in the liver with NASH compared with NAFLD and control (25). In this study, we further demonstrated the result that MT genes (including MT1A, MT1B, and MT1M) were robustly downregulated in NASH compared with NAFL through integrated differential expression analysis with a larger sample size, suggesting a specific role of MT in the later stage of NAFLD.

The involvement of MT in NAFLD has been rarely studied to our knowledge. Loss of *Mt1* and *Mt2* predisposed hepatocytes to apoptosis by activating inflammatory cytokines such as TNF-α (26). Bensellam et al. reported a regulating function of Mt1 in insulin secretion (27), suggesting that Mt1 could play a protective role on hepatocytes by improving insulin resistance. A recent study has shown that Mt1 may mediate the protective effect of IL-22 against hepatocyte death in the CXCL1-induced NASH model (28). Our results in the HFD-induced mouse model clearly showed the impaired function of Mt1 in NASH instead of NAFL. It is still unclear why MT1 is downregulated in NASH. Recently, MT1 was reported to be downregulated in hepatocellular carcinoma and regulated by DNA hypermethylation (29), which may explain why MT1 is downregulated in NASH.

Among MT1 family members, MT1M was reported as a potential target for hepatic cancer therapy (23, 30). The detailed mechanism of the protective effect of MT1M remains unclear. The rescue of lipotoxicity by MT1M overexpression in our *in vitro* experiments was accompanied by obvious downregulation of inflammatory genes, suggesting that the protection may be mediated by inhibiting inflammatory response inside the liver. Interestingly, disorders of metal metabolism (i.e., zinc) have been previously documented in NASH (31). Thus, MT1M may also play roles in NASH by alleviating metal accumulation and related toxicity. In addition, the products of oxidative stress (i.e., oxidized phospholipids) have been proven to accumulate in human and mouse NASH livers (32), and product neutralization by MT1 could ameliorate NASH progression. Taken together, we hypothesize that multiple pathways (anti-inflammation, metal detoxification, anti-oxidation, and improving insulin resistance) might be involved in the protective effect of MT during the transition between NAFL and NASH, and their roles remain to be further investigated and verified by *in vivo* experiments.

There are limitations to our study. First, there is no specific clinical information (metabolic context, age, etc.) to correct the results and no distinction about the degree of NASH (NASH–NASH fibrosis–NASH cirrhosis). Moreover, the samples included in this study are limited, and the role of Mt1 urgently needs to be verified in a larger population. Furthermore, several other genes may also participate in NAFLD progression and await further experimental confirmation.

In conclusion, our integrated analysis identified novel gene signatures underlying the transition from NAFL to NASH. Reduced expression of MT1 may become a potential diagnostic marker for NAFLD progression. Restoring the function of MT1 might be a potential intervention target for blocking the progression from NAFL to NASH and benefiting the prognosis of NAFLD.

## Data availability statement

The datasets presented in this studycan be found in online repositories. The names of the repository/repositories and accession number(s) can be found below: https://www.ncbi.nlm.nih.gov/, GSE48452; https://www.ncbi.nlm.nih.gov/, GSE89632; https://www.ncbi.nlm.nih.gov/, GSE66676; https://www.ncbi.nlm.nih.gov/, GSE126848.

## Ethics statement

This study was reviewed and approved by Laboratory Animal Ethical Committee of Fudan University. Written informed consent was obtained from the owners for the participation of their animals in this study.

## Author contributions

XL, SPZ and YS contribute equally to this work. All authors contributed to the article and approved the submitted version.

## Funding

This work was supported by the Scientific Research Project funded by Shanghai Fifth People’s Hospital, Fudan University (2019WYZD02 to SZa), Talent Development Plan funded by Shanghai Fifth People’s Hospital, Fudan University (No.2020WYRCZY01 to SZa), Natural Science Foundation of Shanghai (19ZR1440200 to JL), and Medical Key Faculty Foundation of Shanghai (ZK2019B15).

## Conflict of interest

The authors declare that the research was conducted in the absence of any commercial or financial relationships that could be construed as a potential conflict of interest.

The reviewer HB declared a shared affiliation with the author(s) to the handling editor at the time of review.

## Publisher’s note

All claims expressed in this article are solely those of the authors and do not necessarily represent those of their affiliated organizations, or those of the publisher, the editors and the reviewers. Any product that may be evaluated in this article, or claim that may be made by its manufacturer, is not guaranteed or endorsed by the publisher.

## Supplementary material

Supplementary Figure 1 Normalization of microarray datasets. **(A)** Normalization of GSE48452 expression profile; **(B)** Normalization of GSE89632 expression profile; **(C)** Normalization of GSE66676 expression profile; Blue represents data before normalization, and red represents data after normalization.

Supplementary Figure 2 Violin plots of the expression level of nine hub genes (GPNMB, SPP1, VCAN, COL1A1, COL1A2, LUM, FAP, FBLN5 and MT1M) between NAFL and healthy livers.

Supplementary Figure 3 Histology pictures of ND, NAFL, and NASH mice. H&E staining of 4 representative mice for each group were shown.

## Abbreviations

AAV, adeno-associated virus; ALT, alanine transaminase; AST, aspartate transferase; ANOVA, analysis of variance; ATCC, American Type Culture Collection; BCA, bicinchoninic acid; BSA, bovine serum albumin; COL1A1, collagen type I alpha 1; COL1A2, collagen type I alpha 2; CDAHFD, choline-deficient, l-amino acid-defined, high fat diet with 0.1% methionine diet; DEG, differentially expressed genes; FAP, fibroblast activation protein; FBLN5, fibulin-5; GEO, Gene Expression Omnibus; GO, gene ontology; GPNMB, transmembrane glycoprotein NMB; H&E, hematoxylin and eosin staining; KEGG, Kyoto Encyclopedia of Genes and Genomes; LUM, lumican; MCP-1, monocyte chemotactic protein 1; MT, metallothionein; NAFLD, non-alcoholic fatty liver disease; NAFL, non-alcoholic fatty liver; NAS, non-alcoholic fatty liver disease activity score; NASH, non-alcoholic steatohepatitis; PPI, protein–protein interaction; RRA, robust rank aggregation; SPP1, secreted phosphoprotein 1; TG, triglyceride; TNF-α, tumor necrosis factor-α; VCAN, versican core protein.
