# Supplementary figures and images for: Integration analysis identifies the role of metallothionein in the progression from hepatic steatosis to steatohepatitis
Source: Front Endocrinol (Lausanne). 2022 Oct 18;13:951093. doi: 10.3389/fendo.2022.951093 (PMC9622801; doi:10.3389/fendo.2022.951093)

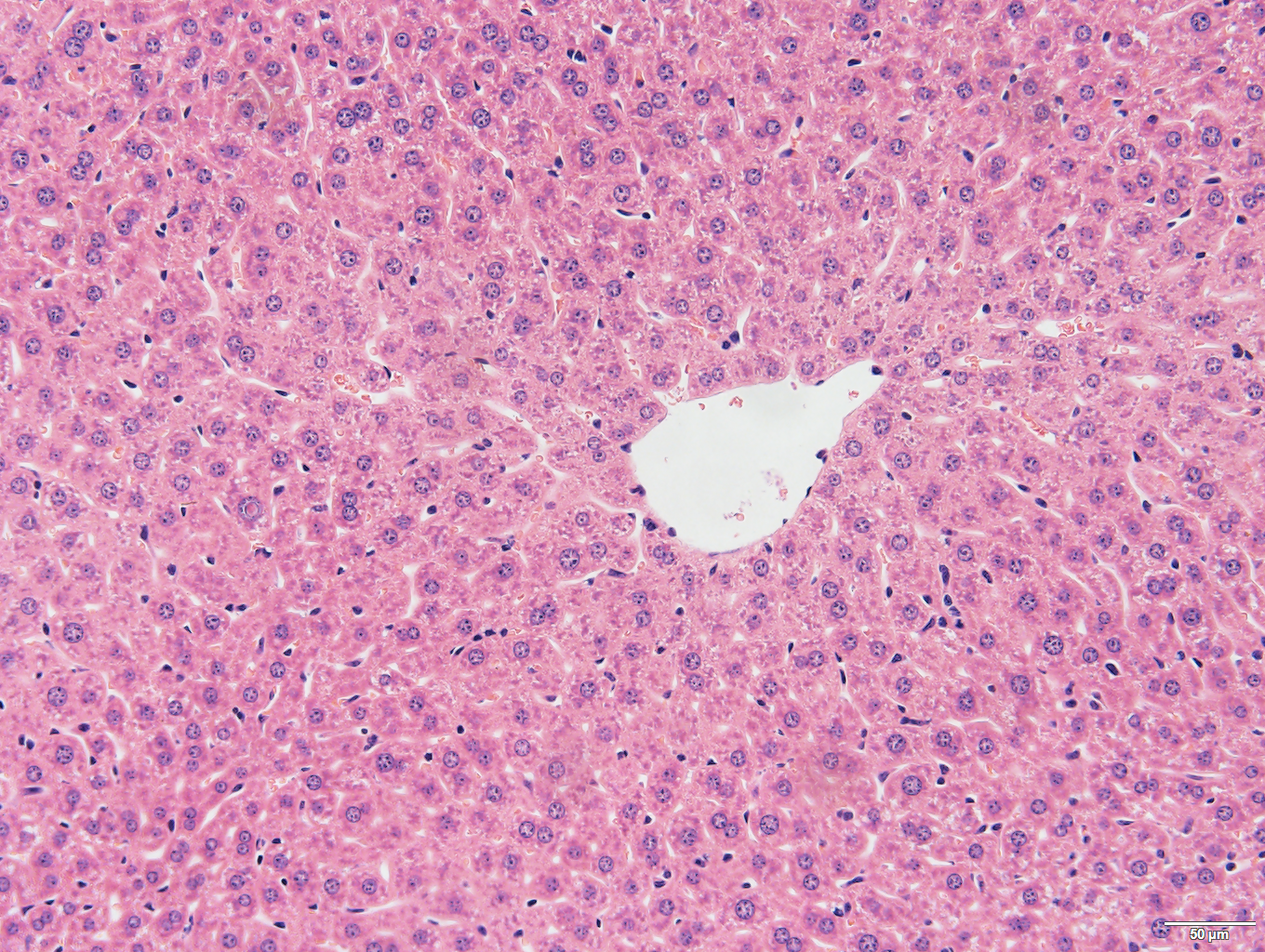

Supplement: Supplementary Figure 1 — Normalization of microarray datasets. (A) Normalization of GSE48452 expression profile; (B) Normalization of GSE89632 expression profile; (C) Normalization of GSE66676 expression profile; Blue represents data before normalization, and red represents data after normalization. [file Image_1.tif]

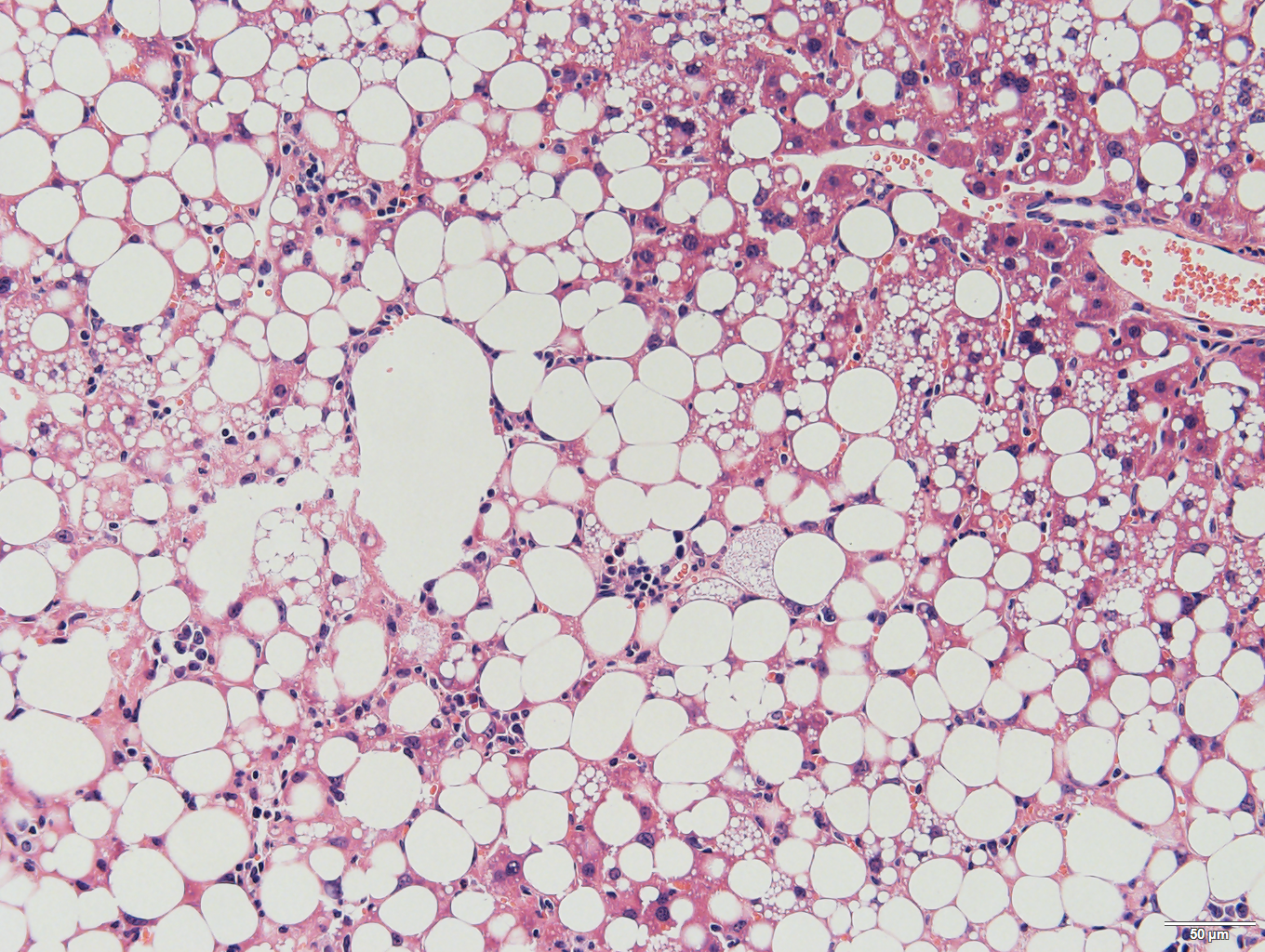

Supplement: Supplementary Figure 2 — Violin plots of the expression level of nine hub genes (GPNMB, SPP1, VCAN, COL1A1, COL1A2, LUM, FAP, FBLN5 and MT1M) between NAFL and healthy livers. [file Image_2.tif]

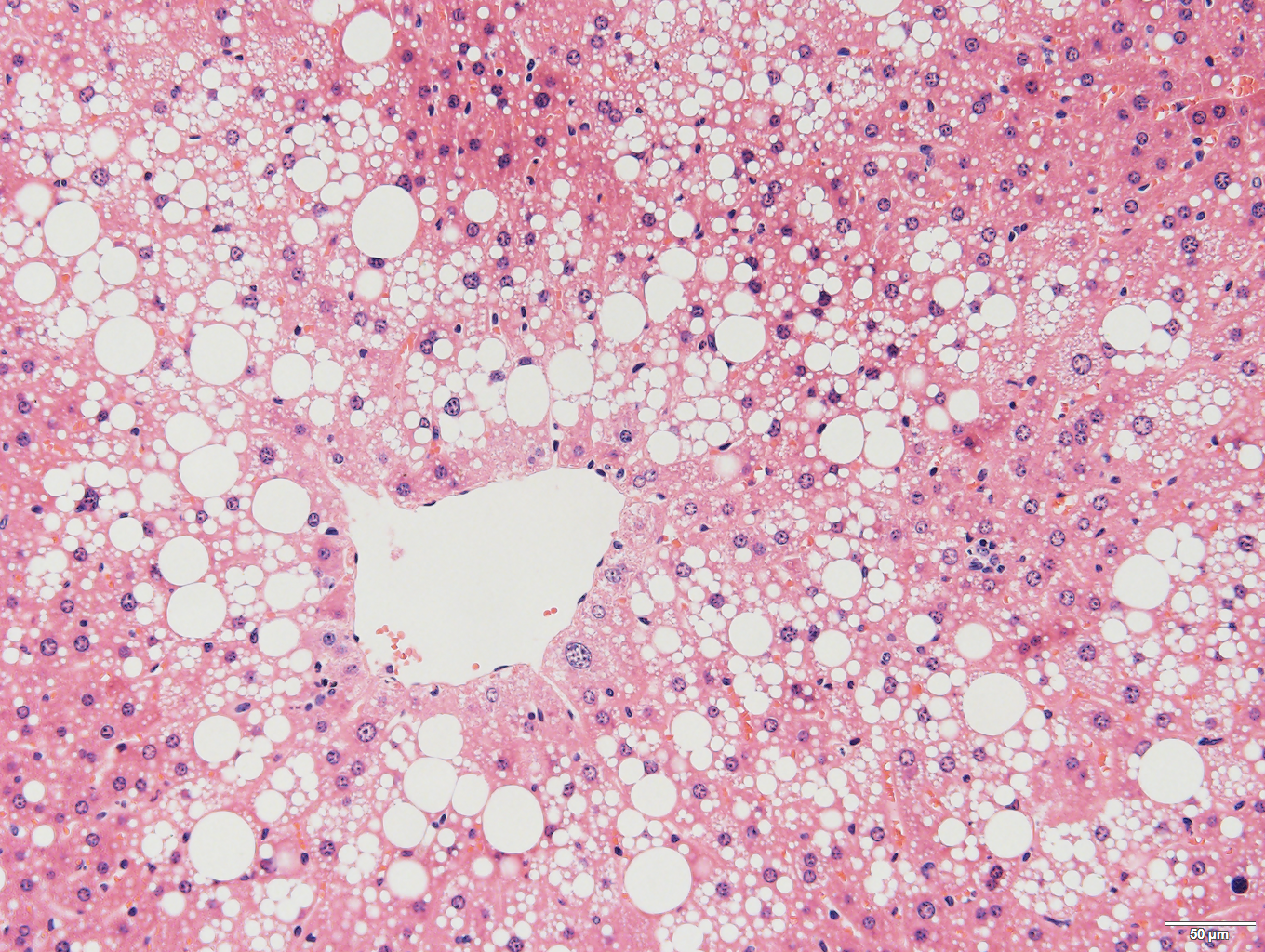

Supplement: Supplementary Figure 3 — Histology pictures of ND, NAFL, and NASH mice. H&E staining of 4 representative mice for each group were shown. [file Image_3.tif]

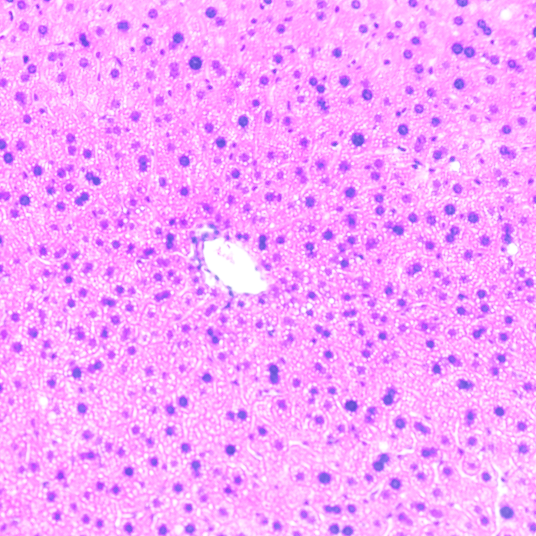

Supplement: Supplementary file 4 [file Image_4.tif]

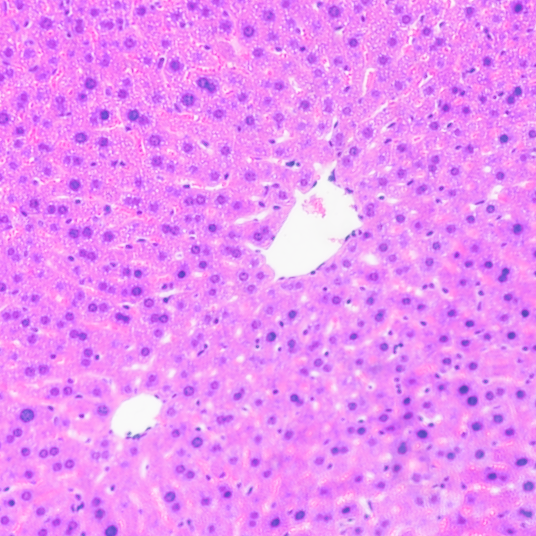

Supplement: Supplementary file 5 [file Image_5.tif]

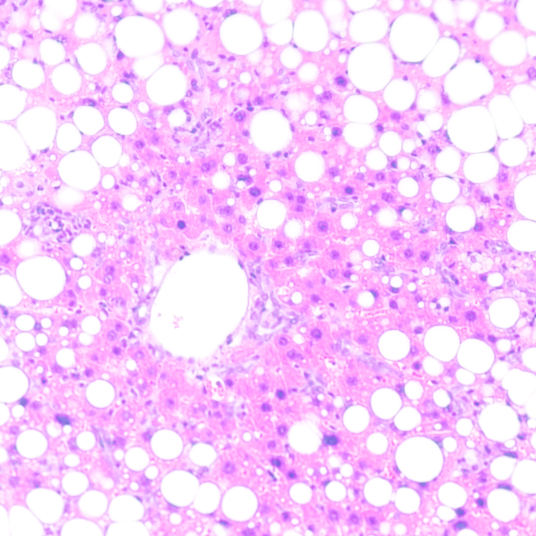

Supplement: Supplementary file 6 [file Image_6.tif]

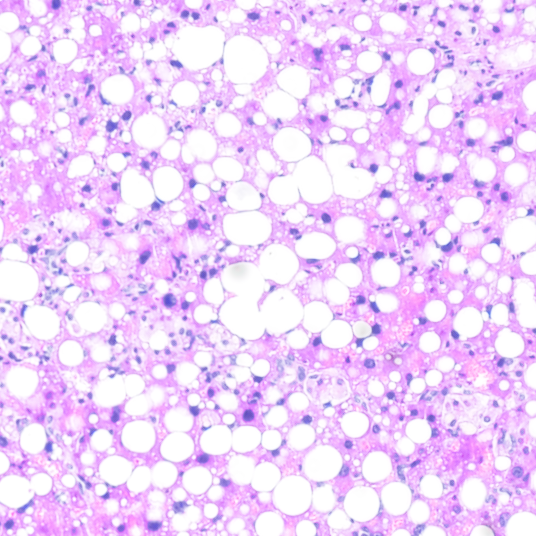

Supplement: Supplementary file 7 [file Image_7.tif]

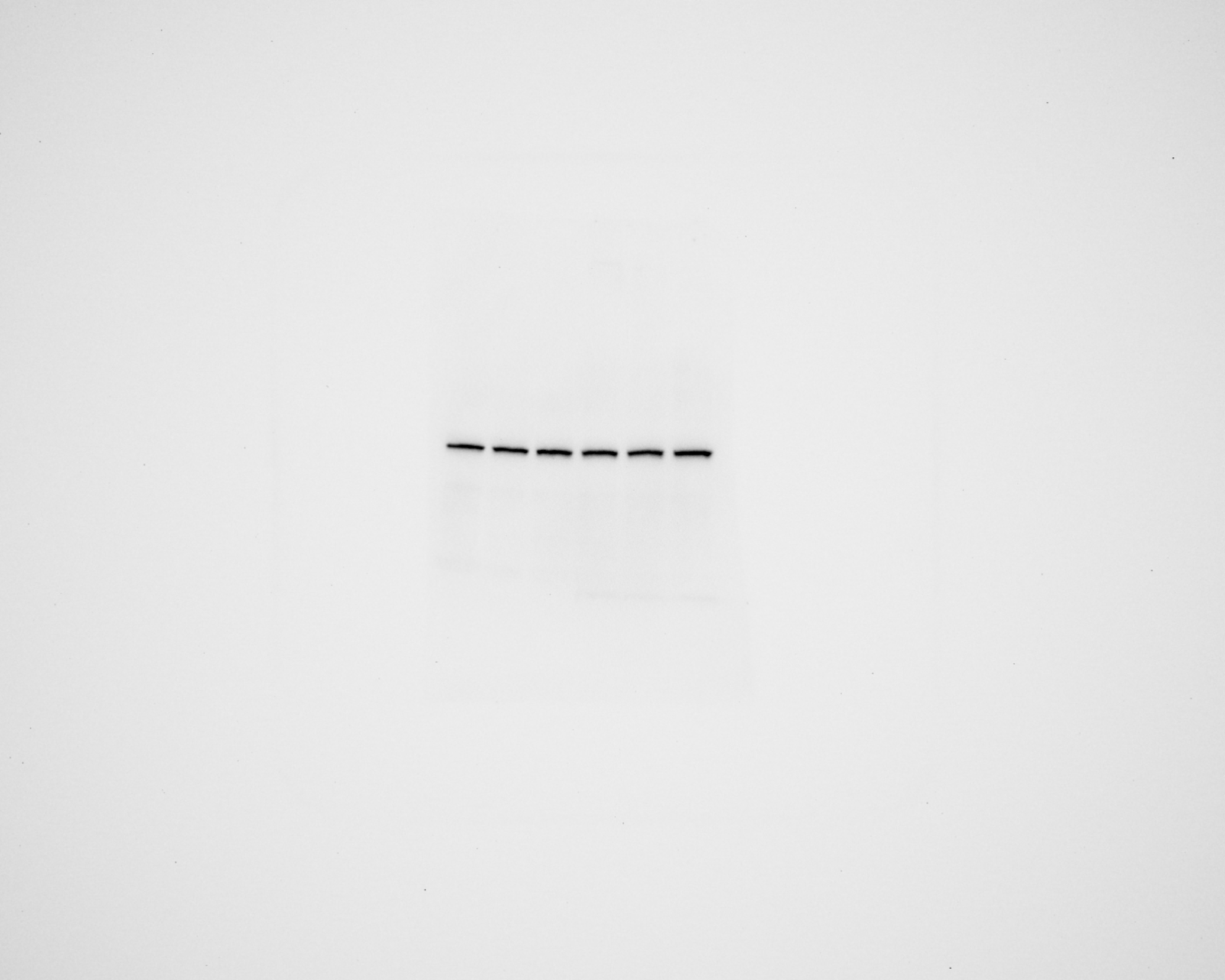

Supplement: Supplementary file 8 [file Image_8.tif]

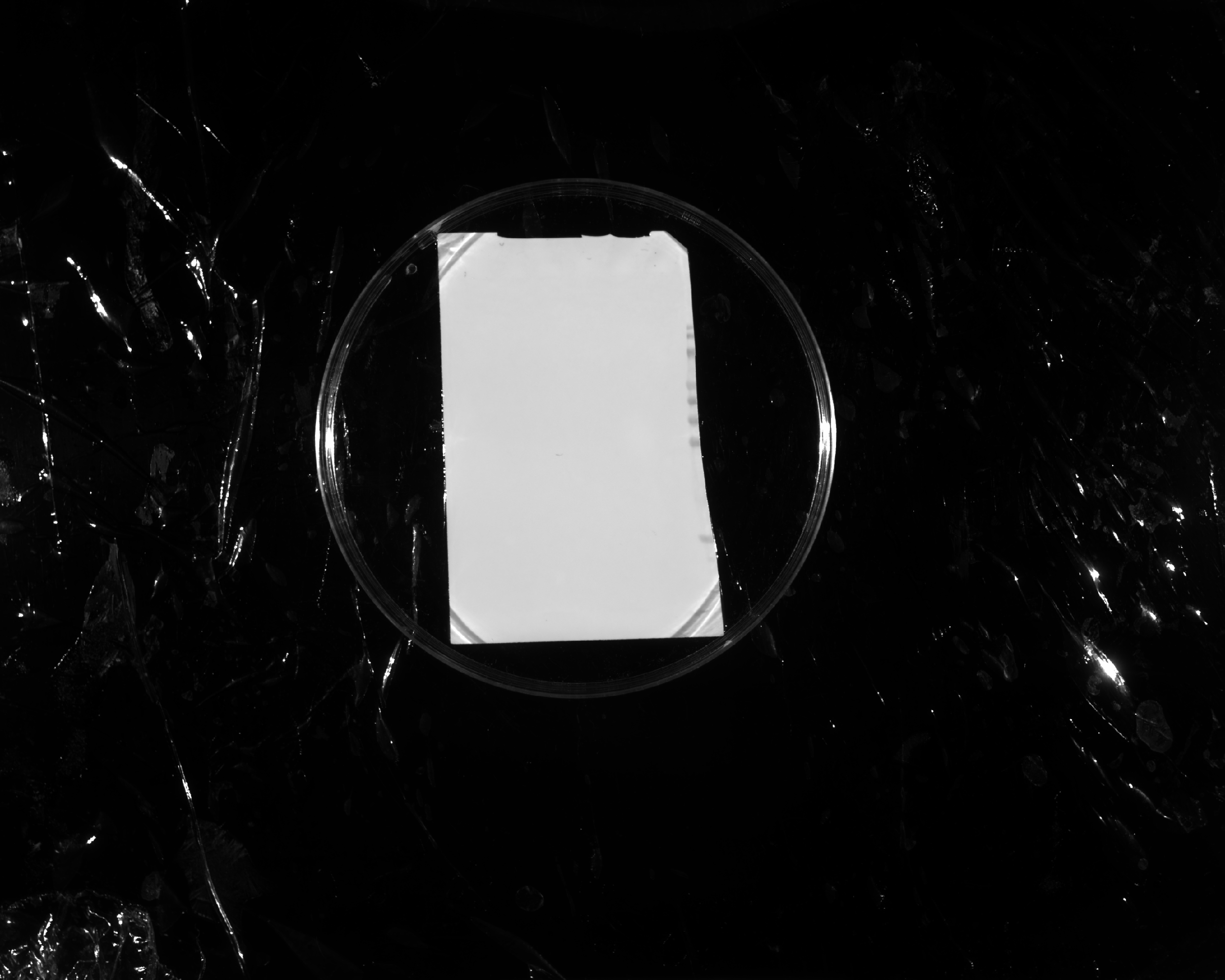

Supplement: Supplementary file 9 [file Image_9.tif]

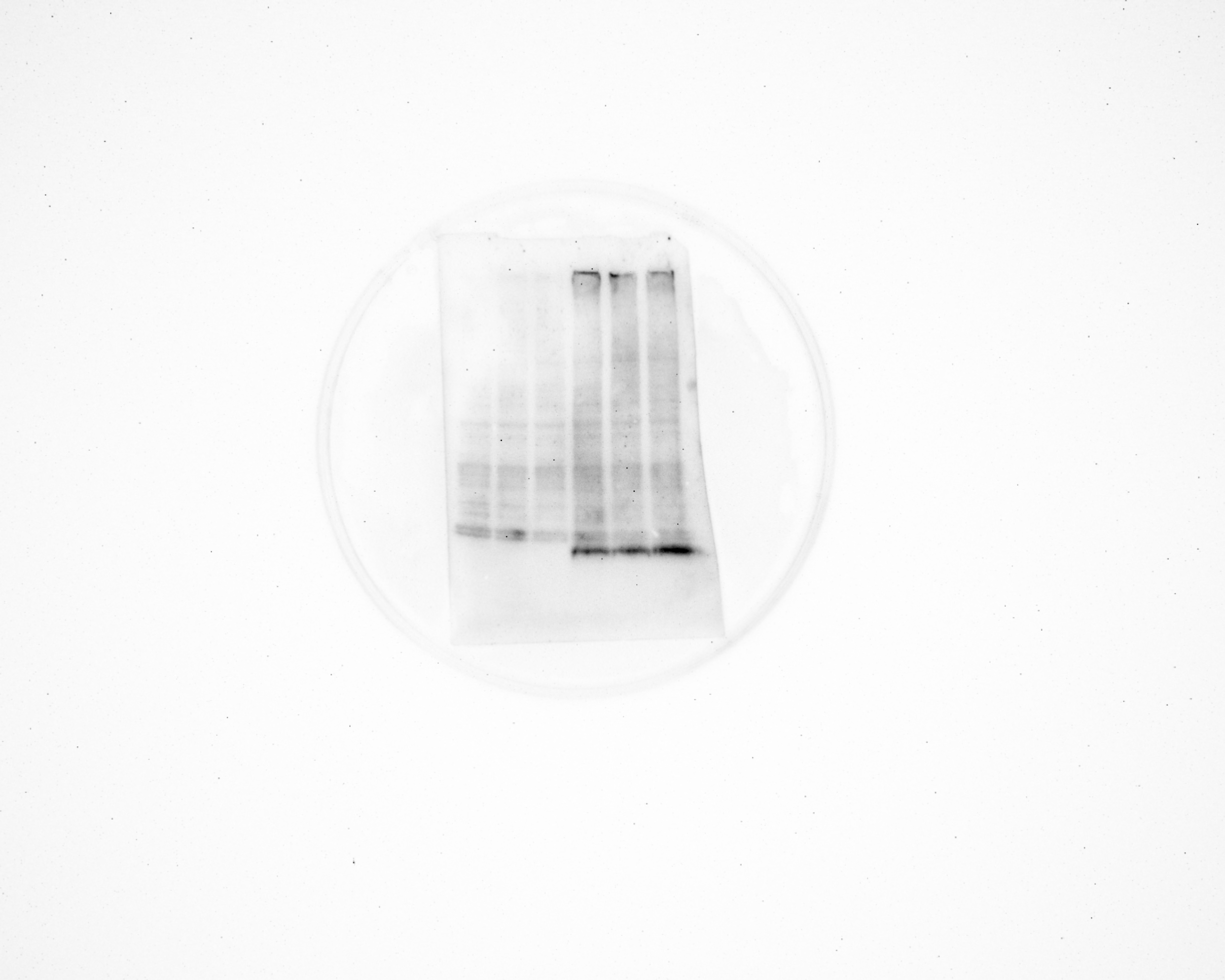

Supplement: Supplementary file 10 [file Image_10.tif]

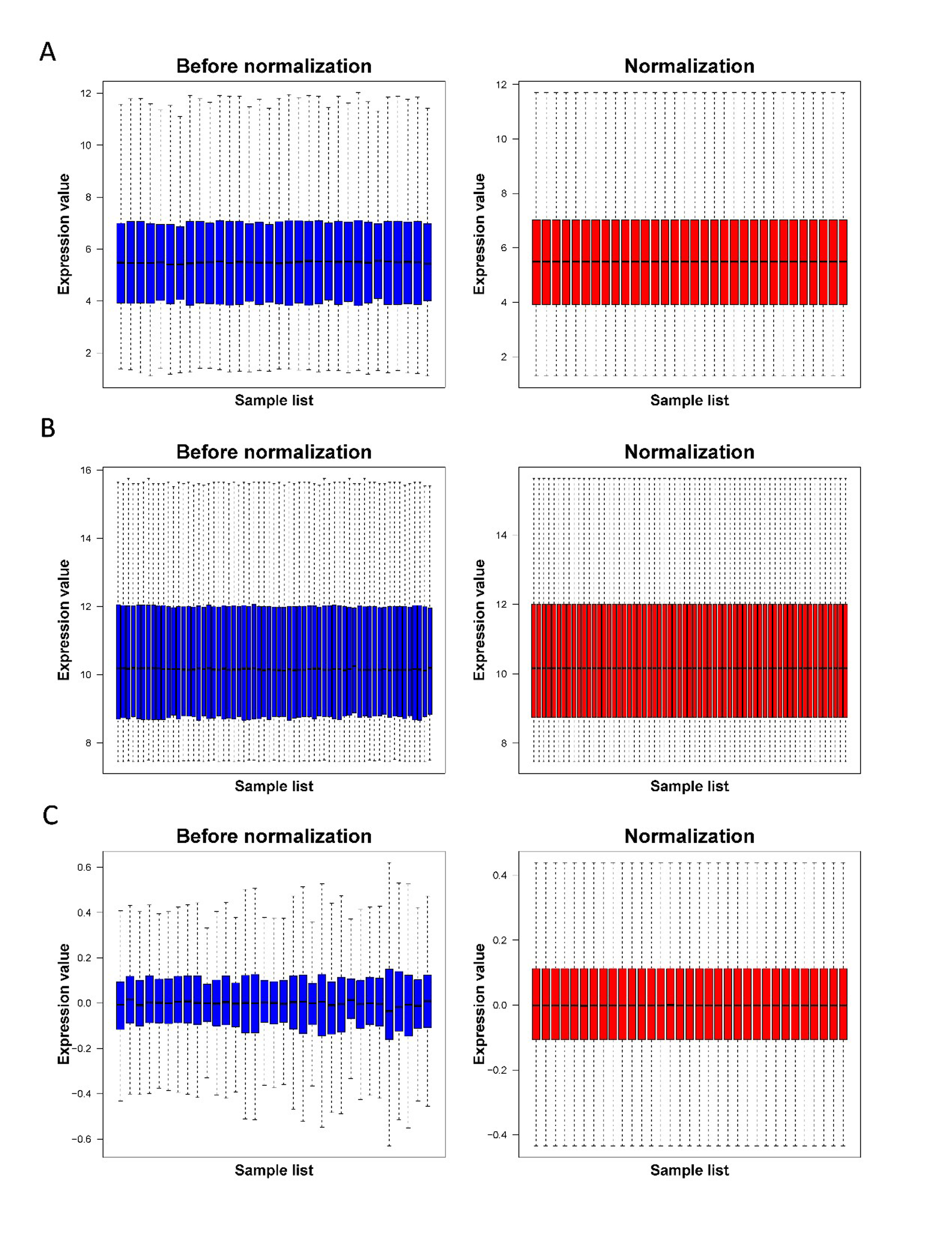

Supplement: Supplementary file 11 [file Image_11.tif]

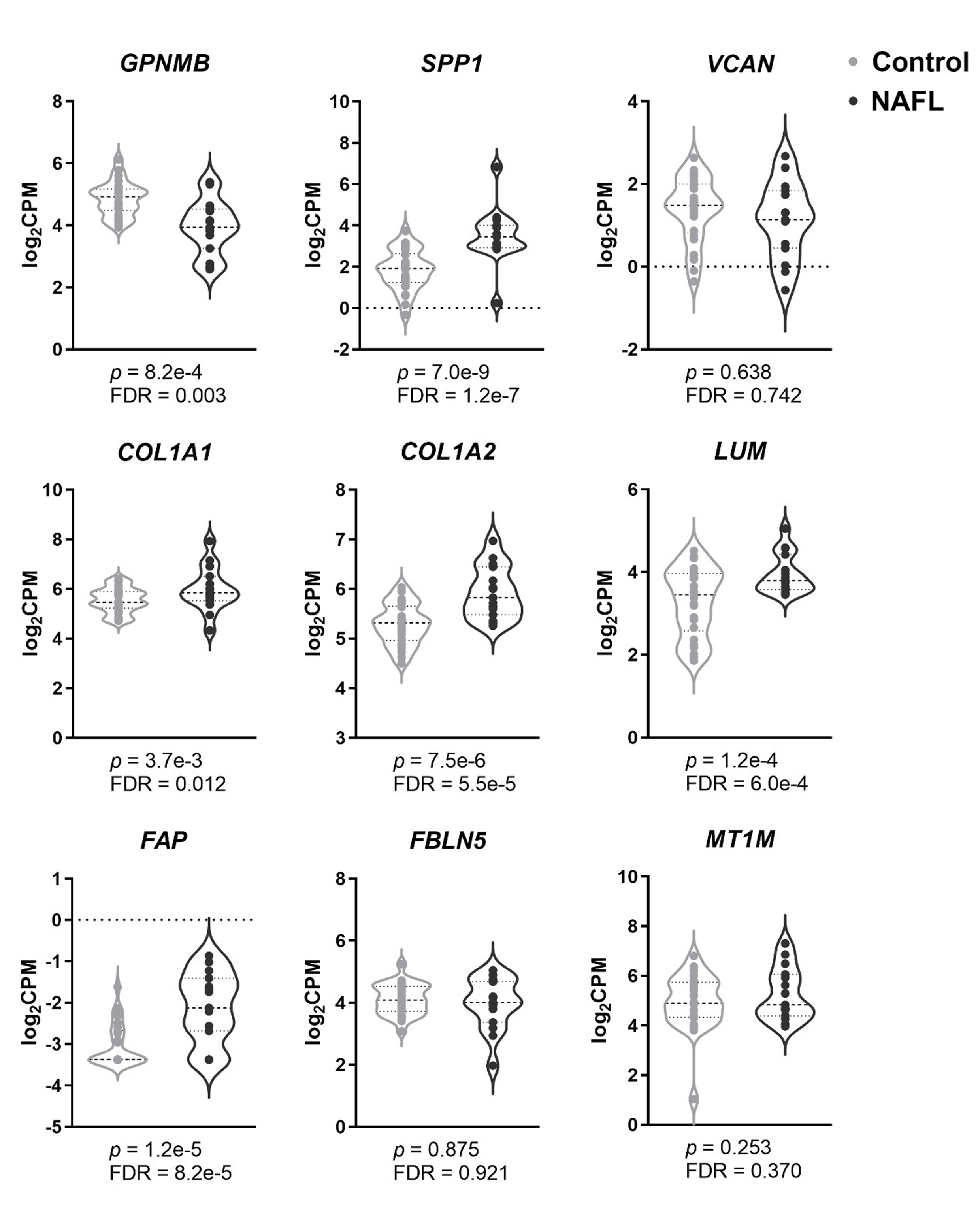

Supplement: Supplementary file 12 [file Image_12.tif]

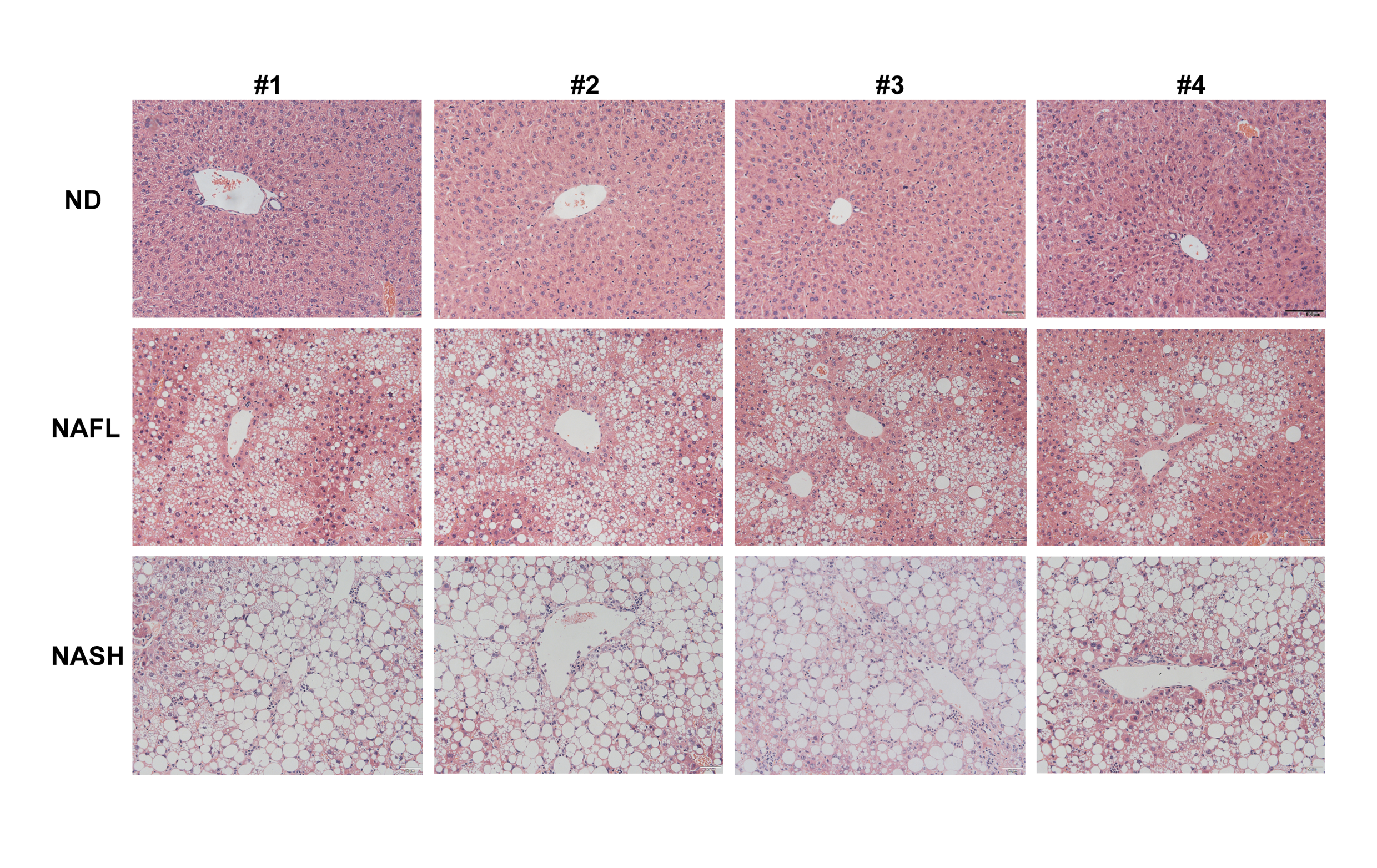

Supplement: Supplementary file 13 [file Image_13.tif]

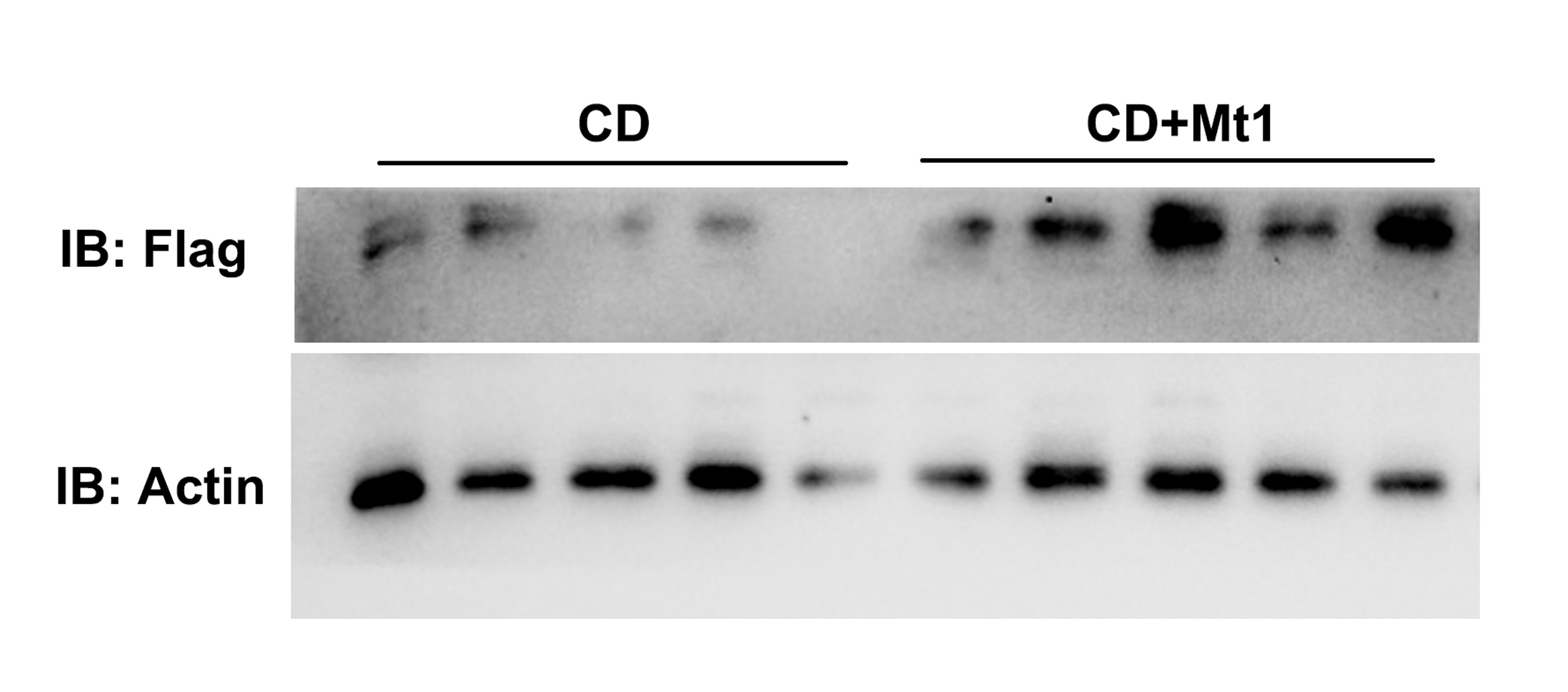

Supplement: Supplementary file 14 [file Image_14.tif]
