# Supplementary material for: Integration analysis identifies the role of metallothionein in the progression from hepatic steatosis to steatohepatitis
Source: Front Endocrinol (Lausanne). 2022 Oct 18;13:951093. doi: 10.3389/fendo.2022.951093 (PMC9622801; doi:10.3389/fendo.2022.951093)
Supplement: Supplementary file 18 [file DataSheet_4.docx]

> setwd("C:/Users/zhong/Desktop/Bio-informatics/NASH Study/RRA4")

> padj=0.05

> logFC=0.58

>

> files=c("GSE48452_limmaTab.txt","GSE89632_limmaTab.txt","GSE66676_limmaTab.txt")

> upList=list()

> downList=list()

> allFCList=list()

> for(i in 1:length(files)){

+ inputFile=files[i]

+ rt=read.table(inputFile,header=T,sep = '\t',quote = '') # 注意文件读取

+ header=unlist(strsplit(inputFile,"_"))

+ downList[[header[1]]]=as.vector(rt[,1])

+ upList[[header[1]]]=rev(as.vector(rt[,1]))

+ fcCol=rt[,1:2]

+ colnames(fcCol)=c("Gene",header[[1]])

+ allFCList[[header[1]]]=fcCol

+ }

>

> mergeLe=function(x,y){

+ merge(x,y,by="Gene",all=T)}

> newTab=Reduce(mergeLe,allFCList)

> rownames(newTab)=newTab[,1]

> newTab=newTab[,2:ncol(newTab)]

> newTab[is.na(newTab)]=0

>

> library(RobustRankAggreg)

> upMatrix = rankMatrix(upList)

> upAR = aggregateRanks(rmat=upMatrix)

> colnames(upAR)=c("Name","Pvalue")

> upAdj=p.adjust(upAR$Pvalue,method="bonferroni")

> upXls=cbind(upAR,adjPvalue=upAdj)

> upFC=newTab[as.vector(upXls[,1]),]

> upXls=cbind(upXls,logFC=rowMeans(upFC))

> write.table(upXls,file="up.xls",sep="\t",quote=F,row.names=F)

> View(upXls)

> upSig=upXls[(upXls$Pvalue<padj & upXls$logFC>logFC),]

>

> View(upSig)

>

>

> downMatrix = rankMatrix(downList)

> downAR = aggregateRanks(rmat=downMatrix)

> colnames(downAR)=c("Name","Pvalue")

> downAdj=p.adjust(downAR$Pvalue,method="bonferroni")

> downXls=cbind(downAR,adjPvalue=downAdj)

> downFC=newTab[as.vector(downXls[,1]),]

> downXls=cbind(downXls,logFC=rowMeans(downFC))

> write.table(downXls,file="down.xls",sep="\t",quote=F,row.names=F)

> downSig=downXls[(downXls$Pvalue<padj & downXls$logFC< -logFC),]

> write.table(downSig,file="downSig.xls",sep="\t",quote=F,row.names=F)

> View(downSig)

>

> allSig = rbind(upSig,downSig)

> colnames(allSig)

[1] "Name" "Pvalue" "adjPvalue" "logFC"

>

> allSig = allSig[,c("Name","logFC")]

> write.table(allSig,file = 'allSign.xls',sep = '\t',quote = F)

>

> View(upSig)

> View(downSig)

> hminput=newTab[c(as.vector(upSig[1:23,1]),as.vector(downSig[1:17,1])),]

> library(pheatmap)

>

> tiff(file="logFC.tiff",width = 15,height = 20,units ="cm",compression="lzw",bg="white",res=400)

> pheatmap(hminput,display_numbers = TRUE,

+ fontsize_row=10,

+ fontsize_col=12,

+ color = colorRampPalette(c("blue", "white", "red"))(50),

+ cluster_cols = FALSE,cluster_rows = FALSE, )

> dev.off()

null device

1
