# Supplementary material for: Integration analysis identifies the role of metallothionein in the progression from hepatic steatosis to steatohepatitis
Source: Front Endocrinol (Lausanne). 2022 Oct 18;13:951093. doi: 10.3389/fendo.2022.951093 (PMC9622801; doi:10.3389/fendo.2022.951093)
Supplement: Supplementary file 20 [file DataSheet_6.docx]

setwd("C:/Users/zhong/Desktop/Bio-informatics/NASH Study/GSE126848")

> dir.create("result")

> library("DESeq2")

载入需要的程辑包：S4Vectors

载入需要的程辑包：stats4

载入需要的程辑包：BiocGenerics

载入需要的程辑包：parallel

载入程辑包：‘BiocGenerics’

The following objects are masked from ‘package:parallel’:

clusterApply, clusterApplyLB, clusterCall, clusterEvalQ, clusterExport, clusterMap,

parApply, parCapply, parLapply, parLapplyLB, parRapply, parSapply, parSapplyLB

The following objects are masked from ‘package:stats’:

IQR, mad, sd, var, xtabs

The following objects are masked from ‘package:base’:

anyDuplicated, append, as.data.frame, basename, cbind, colnames, dirname, do.call,

duplicated, eval, evalq, Filter, Find, get, grep, grepl, intersect, is.unsorted, lapply,

Map, mapply, match, mget, order, paste, pmax, pmax.int, pmin, pmin.int, Position, rank,

rbind, Reduce, rownames, sapply, setdiff, sort, table, tapply, union, unique, unsplit,

which, which.max, which.min

载入程辑包：‘S4Vectors’

The following object is masked from ‘package:base’:

expand.grid

载入需要的程辑包：IRanges

载入程辑包：‘IRanges’

The following object is masked from ‘package:grDevices’:

windows

载入需要的程辑包：GenomicRanges

载入需要的程辑包：GenomeInfoDb

载入需要的程辑包：SummarizedExperiment

载入需要的程辑包：Biobase

Welcome to Bioconductor

Vignettes contain introductory material; view with 'browseVignettes()'. To cite

Bioconductor, see 'citation("Biobase")', and for packages 'citation("pkgname")'.

载入需要的程辑包：DelayedArray

载入需要的程辑包：matrixStats

载入程辑包：‘matrixStats’

The following objects are masked from ‘package:Biobase’:

anyMissing, rowMedians

载入需要的程辑包：BiocParallel

载入程辑包：‘DelayedArray’

The following objects are masked from ‘package:matrixStats’:

colMaxs, colMins, colRanges, rowMaxs, rowMins, rowRanges

The following objects are masked from ‘package:base’:

aperm, apply, rowsum

> library("rlang")

载入程辑包：‘rlang’

The following object is masked from ‘package:Biobase’:

exprs

> library(tidyverse)

-- **Attaching packages** --------------------------------------- tidyverse 1.3.0 --

√ ggplot2 3.3.0 √ purrr 0.3.3

√ tibble 2.1.3 √ dplyr 0.8.5

√ tidyr 1.0.0 √ stringr 1.4.0

√ readr 1.3.1 √ forcats 0.4.0

-- **Conflicts** ------------------------------------------ tidyverse_conflicts() --

x purrr::%@%() masks rlang::%@%()

x purrr::as_function() masks rlang::as_function()

x dplyr::collapse() masks IRanges::collapse()

x dplyr::combine() masks Biobase::combine(), BiocGenerics::combine()

x dplyr::count() masks matrixStats::count()

x dplyr::desc() masks IRanges::desc()

x tidyr::expand() masks S4Vectors::expand()

x rlang::exprs() masks Biobase::exprs()

x dplyr::filter() masks stats::filter()

x dplyr::first() masks S4Vectors::first()

x purrr::flatten() masks rlang::flatten()

x purrr::flatten_chr() masks rlang::flatten_chr()

x purrr::flatten_dbl() masks rlang::flatten_dbl()

x purrr::flatten_int() masks rlang::flatten_int()

x purrr::flatten_lgl() masks rlang::flatten_lgl()

x purrr::flatten_raw() masks rlang::flatten_raw()

x purrr::invoke() masks rlang::invoke()

x dplyr::lag() masks stats::lag()

x purrr::list_along() masks rlang::list_along()

x purrr::modify() masks rlang::modify()

x ggplot2::Position() masks BiocGenerics::Position(), base::Position()

x purrr::prepend() masks rlang::prepend()

x purrr::reduce() masks GenomicRanges::reduce(), IRanges::reduce()

x dplyr::rename() masks S4Vectors::rename()

x purrr::simplify() masks DelayedArray::simplify()

x dplyr::slice() masks IRanges::slice()

x purrr::splice() masks rlang::splice()

>

> mycounts<-read.csv("GSE126848_series_matrix (sample_title).csv",header = T,row.names = 1)

> View(mycounts)

> condition <- factor(c(rep("control",15),rep("NASH",16)), levels = c("control","NASH"))

> colData =read.csv("colData.csv",header = T,row.names = 1)

> View(colData)

> dds <- DESeqDataSetFromMatrix(mycounts, colData, design= ~ condition)

> dds <- DESeq(dds)

estimating size factors

estimating dispersions

gene-wise dispersion estimates

mean-dispersion relationship

final dispersion estimates

fitting model and testing

-- replacing outliers and refitting for 168 genes

-- DESeq argument 'minReplicatesForReplace' = 7

-- original counts are preserved in counts(dds)

estimating dispersions

fitting model and testing

>

> View(dds)

> tiff(file="result/dispersion_plot.tiff",width = 16,height =16 ,

+ units ="cm",compression="lzw",bg="white",res=300)

> plotDispEsts(dds,main = "Dispersion plot")

> dev.off()

null device

1

>

> res = results(dds, contrast=c("condition", "NASH", "control"))

> View(res)

> res = res[order(res$pvalue),]

> res=subset(res,res$log2FoldChange!="<NA>")

> write.csv(res,file="result/All_results.csv")

> diff = subset(res,res$padj<0.05&abs(res$log2FoldChange)>=1)

> write.table(diff,"result/diff_results.xls",sep = "\t",quote = F,row.names = F)

>

> View(diff)

> View(diff)

> library("biomaRt")

> listMarts()

biomart version

1 ENSEMBL_MART_ENSEMBL Ensembl Genes 99

2 ENSEMBL_MART_MOUSE Mouse strains 99

3 ENSEMBL_MART_SNP Ensembl Variation 99

4 ENSEMBL_MART_FUNCGEN Ensembl Regulation 99

> my_mart <- useMart(biomart = "ENSEMBL_MART_ENSEMBL", dataset = "hsapiens_gene_ensembl",version = "Ensembl Genes 99")

> All_results_ENSID <- read.csv(file = "All_results_ENSID.csv",header = TRUE, sep = ",")

> View(All_results_ENSID)

> convert_list <- getBM(attributes=c("ensembl_gene_id","hgnc_symbol"),filters = "ensembl_gene_id",values=All_results_ENSID$ENSID, mart= my_mart,uniqueRows=F)

Batch submitting query [===============>----------------------------------------] 28% eta: 2m错误: biomaRt has encountered an unexpected server error.

Consider trying one of the Ensembl mirrors (for more details look at ?useEnsembl)

> convert_list <- getBM(attributes=c("ensembl_gene_id","hgnc_symbol"),filters = "ensembl_gene_id",values=All_results_ENSID$ENSID, mart= my_mart,uniqueRows=F)

Batch submitting query [==================>-------------------------------------] 33% eta: 23s错误: biomaRt has encountered an unexpected server error.

Consider trying one of the Ensembl mirrors (for more details look at ?useEnsembl)

> ?useEnsembl

> convert_list <- getBM(attributes=c("ensembl_gene_id","hgnc_symbol"),filters = "ensembl_gene_id",values=All_results_ENSID$ENSID, mart= my_mart,uniqueRows=F)

Batch submitting query [==============================================>---------] 83% eta: 26s错误: biomaRt has encountered an unexpected server error.

Consider trying one of the Ensembl mirrors (for more details look at ?useEnsembl)

> convert_list <- getBM(attributes=c("ensembl_gene_id","hgnc_symbol"),filters = "ensembl_gene_id",values=All_results_ENSID$ENSID, mart= my_mart,uniqueRows=F)

Batch submitting query [=================================================>------] 89% eta: 3s错误: biomaRt has encountered an unexpected server error.

Consider trying one of the Ensembl mirrors (for more details look at ?useEnsembl)

> convert_list <- getBM(attributes=c("ensembl_gene_id","hgnc_symbol"),filters = "ensembl_gene_id",values=All_results_ENSID$ENSID, mart= my_mart,uniqueRows=F)

> View(convert_list)

> names(convert_list)<-c("ENSID","Symbol")

> View(All_results_ENSID)

> All_results_Symbol <- join(All_results_ENSID,convert_list, by = "ENSID")

Error in join(All_results_ENSID, convert_list, by = "ENSID") :

没有"join"这个函数

> library(plyr)

-------------------------------------------------------------------------------------------------------

You have loaded plyr after dplyr - this is likely to cause problems.

If you need functions from both plyr and dplyr, please load plyr first, then dplyr:

library(plyr); library(dplyr)

-------------------------------------------------------------------------------------------------------

载入程辑包：‘plyr’

The following objects are masked from ‘package:dplyr’:

arrange, count, desc, failwith, id, mutate, rename, summarise, summarize

The following object is masked from ‘package:purrr’:

compact

The following object is masked from ‘package:matrixStats’:

count

The following object is masked from ‘package:IRanges’:

desc

The following object is masked from ‘package:S4Vectors’:

rename

> All_results_Symbol <- join(All_results_ENSID,convert_list, by = "ENSID")

> View(All_results_Symbol)

> write.csv(All_results_Symbol,file = "all_diff.csv")

> up_diff <- All_results_Symbol[with(All_results_Symbol, (log2FoldChange>= 1 & padj < 0.05 )), ]

> View(up_diff)

> write.csv(up_diff,file = "up_diff_Sig.csv")

> down_diff <- All_results_Symbol[with(All_results_Symbol, (log2FoldChange<= 1 & padj < 0.05 )), ]

> View(down_diff)

> write.csv(down_diff,file = "down_diff_Sig.csv")

> down_diff <- All_results_Symbol[with(All_results_Symbol, (log2FoldChange <= -1 & padj < 0.05 )), ]

> View(down_diff)

> All_results_Symbol <- All_results_Symbol[,-0]

Error in .subset2(x, i, exact = exact) : 下标出界

> View(All_results_Symbol)

> View(All_results_ENSID)

> all_diff1 <- read.csv(file = "all_diff1.csv",header = TRUE, sep = ",")

> View(all_diff1)

> up_diff <- all_diff1[with(all_diff1, (log2FoldChange>= 1 & pvalue < 0.05 )), ]

> View(up_diff)

> write.csv(up_diff,file = "up_diff_Sig.csv")

> down_diff <- all_diff1[with(all_diff1, (log2FoldChange <= -1 & pvalue < 0.05 )), ]

> View(down_diff)

> write.csv(down_diff,file = "down_diff_Sig.csv")
